# Supplementary material for: Community Based Interventions for Problematic Substance Use in Later Life: A Systematic Review of Evaluated Studies and Their Outcomes
Source: Int J Environ Res Public Health. 2020 Oct 30;17(21):7994. doi: 10.3390/ijerph17217994 (PMC7663344; doi:10.3390/ijerph17217994)
Supplement: Supplementary file 1 [file ijerph-17-07994-s001.pdf]

Supplementary Materials

Table S1. Full Search Strategy

1 BioMed Central<br>Keyword Search;<br>Participants: Old* people or old* adults or elderly or ageing or aging or geriat* or geron* or mature AND<br>Topic: Addict* "Problematic substance use" or "substance misuse" or "alcohol misuse" or "alcoholism" or "drug misuse" or "drug abuse" or "alcohol abuse" or alcohol or "other drugs" or addiction or "dual diagnosis" or "drug depend*" or "alcohol depend*"
2 International Bibliography of the Social Sciences<br>Keyword Search;<br>Participants: "Old* people" or "old* adults" or elderly or ageing or aging or geriat* or geron* or mature AND<br>Topic: Addict* "Problematic substance use" or "substance misuse" or "alcohol misuse" or "alcoholism" or "drug misuse" or "drug abuse" or "alcohol abuse" or alcohol or "other drugs" or addiction or "dual diagnosis" or "drug depend*" or "alcohol depend*"
3 NICE Evidence<br>Keyword Search;<br>Participants: "Old* people" or "old* adults" or elderly or ageing or aging or geriat* or geron* or mature AND<br>Topic: Addict* "Problematic substance use" or "substance misuse" or "alcohol misuse" or "alcoholism" or "drug misuse" or "drug abuse" or "alcohol abuse" or alcohol or "other drugs" or addiction or "dual diagnosis" or "drug depend*" or "alcohol depend*"
4 OVID Nursing<br>Keyword and MeSH Heading Search<br>**NOTE;** MESH headings in bold below<br>Participants: Old* people or old* adults or elderly or ageing or aging or geriat* or geron* or mature or "aged, **80 and over" or "aged**" AND<br>Topic: Addict* or alcohol misuse or alcoholism or drug misuse or drug abuse or alcohol abuse or alcohol or other drugs or addiction or dual diagnosis or drug depend* or alcohol depend* or **substance use or substance use disorders**
5 EBSCO Platform—CINAHL<br>Keyword and MeSH Heading Search<br>**NOTE;** MESH headings in bold below<br>Participants: "Old* people" or "old* adults" or elderly or ageing or aging or geriat* or geron* or mature or **"aged, 80 and over" or "aged"** AND<br>Topic: Addict* "Problematic substance use" or "substance misuse" or "alcohol misuse" or "alcoholism" or "drug misuse" or "drug abuse" or "alcohol abuse" or alcohol or "other drugs" or addiction or "dual diagnosis" or "drug depend*" or "alcohol depend*" or **"Substance dependence" or "substance addiction consequences" or "alcoholism" or "street drugs"**

6 EBSCO Platform—MEDLINE

Keyword and MeSH Heading Search

NOTE; MESH headings in bold below

Participants: “Old* people” or “old* adults” or elderly or ageing or aging or geriat* or geron* or mature or **“aged, 80 and over” or “aged”** AND

Topic: Addict* “Problematic substance use” or “substance misuse” or “alcohol misuse” or “alcoholism” or “drug misuse” or “drug abuse” or “alcohol abuse” or “alcohol or other drugs” or narcotics or addiction or “dual diagnosis” or “drug depend*” or “alcohol depend*” **or “opium dependence” or substance-related disorders” or “cocaine related disorders” or Heroin dependence” or “alcoholism” or “amphetamine related disorders” or “drug users”**

---

7 EBSCO Platform—PsycINFO

Keyword and MeSH Heading Search

NOTE; MESH headings in bold below

Participants: “Old* people” or “old* adults” or elderly or ageing or aging or geriat* or geron* or mature or “aging” or **“aged (attitudes toward)” or “aging (attitudes toward)”** AND

Topic: Addict* “Problematic substance use” or “substance misuse” or “alcohol misuse” or “alcoholism” or “drug misuse” or “drug abuse” or “alcohol abuse” or alcohol or “other drugs” or addiction or “dual diagnosis” or “drug depend*” or “alcohol depend*” or **“addiction” or “substance related and addictive disorders” or “drugs” or “substance use disorder” or “dual diagnosis” or alcoholism**

---

8 Cochrane

Keyword and MeSH Heading Search

NOTE; MESH headings in bold below

Participants: “Old* people” or “old* adults” or elderly or ageing or aging or geriat* or geron* or mature or **“aged”** AND

Topic: Addict* “Problematic substance use” or “substance misuse” or “alcohol misuse” or “alcoholism” or “drug misuse” or “drug abuse” or “alcohol abuse” or alcohol or “other drugs” or addiction or “dual diagnosis” or “drug depend*” or “alcohol depend*” or **“drug users” or “substance-related disorders” or alcoholism**

---

9 Social Care Online

Keyword Search

Participants: “Old* people” or “old* adults” or elderly or ageing or aging or geriat* or geron* or mature AND

Topic: Addict* “Problematic substance use” or “substance misuse” or “alcohol misuse” or “alcoholism” or “drug misuse” or “drug abuse” or “alcohol abuse” or alcohol or “other drugs” or addiction or “dual diagnosis” or “drug depend*” or “alcohol depend*”

Table S2. The Participants, Interventions, Comparisons, Outcome, Study Design (PICOS) Eligibility Criteria (Joanna Briggs Institute, 2001).

**Participants:** "Old* people" or "old* adults" or elderly or ageing or aging or geriat* or geron* or mature or "aged, 80 and over" or "aged" AND
**Topic:** Addict* "Problematic substance use" or "substance misuse" or "alcohol misuse" or "alcoholism" or "drug misuse" or "drug abuse" or "alcohol abuse" or AOD or alcohol or "other drugs" or polypharmacy or "prescription drugs" or "non-prescription drugs" or narcotics or addiction or "dual diagnosis" or "drug depend*" or "alcohol depend*" or "Substance dependence" or "substance addiction consequences" or "alcoholism" or "street drugs" AND
**Interventions:** "Self-help" or "mutual aid" or "alcoholics anonymous" or "narcotics anonymous" or "cocaine anonymous" or "alcohol service" or "drug service" or "drug and alcohol service" or "addiction service" or "substance misuse service" or "social care" or "health care" or "community care" or "community intervention" or "peer support" or "drug treatment" or "alcohol treatment" or "alcohol prevention" or "drug prevention" or "drug intervention" or "alcohol intervention" or "substance misuse intervention" or "recovery program*" or rehab* or program* or detox or "detox service" or "needle exchange" or "contingency management" or "methadone maintenance therapy" or "methadone replacement therapy" or "opioid replacement therapy" or "substitution therapy" or "residential rehab" or "men's shed" or "relapse prevention" or "motivational interviewing" or "dialectic behavioural therapy" or "cognitive behavioural therapy" or "substance use treatment: overdose" or "substance use treatment: alcohol withdrawal" or "substance use treatment: drug withdrawal" or " substance use treatment" or "substance use rehabilitation programs" or "treatment outcomes"
**Comparators:** Other age groups and general populations and mental health AND
**Outcomes:** Abstinence, abstention, harm reduction, reduced drinking, sobriety, drug free, wellbeing, improved relationships, interpersonal, reduction in use.
**Study design:** Pre-/post deign, Quasi-experimental desbign, RCT, qualitative study, cohort study, mixed methods.
**NOTE:** Interventions, comparators and outcomes search terms not used due to narrowing search results. A broader search was carried out focusing on participant and topic search terms to ensure we captured all relevant studies.

Table S3. MERSQI Assessment Scores

Domain	MERSQI Item	Study #	1	2	3	4	5	6	7	8	9	10	11	12	13	14	15	16	17	18	19
Study Design	**1. Study Design**	**Score**																			
	Single group cross-sectional or single group post-test only	**1**						1										1	1		
	Single group PP	**1**			1				1												
	Nonrandomized, two group	**2**										2		2		2	2			2	
	Randomized control trial	**3**		3			3			3	3	3	3		3						3
**Sampling**	**2. Number of Institutions Studied**																				
	1	**0.5**	0.5	0.5	0.5		0.5	0.5				0.5	0.5			0.5	0.5	0.5	0.5		
	2	**1**				1								1							
	>2	**1.5**							1.5	1.5	1.5				1.5					1.5	1.5
	**3. Response Rate**																				
	Not applicable	**0**																	0		
	<50 or not reported	**0.5**						0.5				0.5	0.5	0.5				0.5			
	50–74	**1**																			
	>75	**1.5**	1.5	1.5	1.5	1.5	1.5		1.5	1.5	1.5				1.5	1.5	1.5			1.5	1.5
Type of Data	**4. Type of Data**																				
	Assessment by participant	**1**			1			1	1												
	Objective measurement	**3**	3	3		3	3			3	3	3	3	3	3	3	3	3	3	3	3
Validity of evaluation instrument	**5. Internal Structure**																				
	Not applicable																				
	Not reported	**0**	0	0	0			0											0		
	Reported	**1**				1	1		1	1	1	1	1	1	1	1	1	1		1	1
	**6. Content**																				
	Not applicable																				
	Not reported	**0**																	0		
	Reported	**1**	1	1	1	1	1	1	1	1	1	1	1	1	1	1	1	1		1	1
	**7. Relationships to variables**																				
	Not applicable																				
	Not reported	**0**																	0		
	Reported	**1**	1	1	1	1	1	1	1	1	1	1	1	1	1	1	1	1		1	1
**Data Analysis**	**8. Appropriateness of analysis**																				
	Data analysis inappropriate for study design or type of data	**0**																			
	Data analysis appropriate for	**1**	1	1	1	1	1	1	1	1	1	1	1	1	1	1	1	1	1	1	1

	study design or type of data																				
	**9. Complexity of analysis**																				
	Descriptive analysis only	**1**	1		1			1						1				1	1		
	Beyond descriptive analysis	**2**		2		2	2		2	2	2	2	2		2	2	2			2	2
**Outcomes**	**10. Outcomes**																				
	Satisfaction, attitudes, perceptions, opinions, facts	**1**						1						1					1		
	Knowledge, skills	**1.5**			1.5													1.5			
	Behaviours	**2**																			
	Patient/health care outcomes	**3**	3	3		3	3		3	3	3	3	3		3	3	3			3	3
**Total Score**			**12**	**16**	**9.5**	**14.5**	**17**	**8**	**14**	**18**	**18**	**18**	**16**	**12.5**	**18**	**16**	**16**	**11.5**	**7.5**	**17**	**18**

Table S4. Quality Assessment of RCT Studies Using CASP

RCT included studies	2. Barnes et al. (2016)	5. D'Agostino et al. (2006)	8. Fleming et al. (1999)	9. Gottlieb Hansen et al. (2012)	14. Oslin et al. (2004)	20. Watson et al. (2013)
1. Did the trial address a clearly focused issue?	Yes	Yes	Yes	Yes	Yes	Yes
2. Was the assignment of patients to treatments randomised?	Yes	Yes	Yes	Yes	Yes	Yes
3. Were all of the patients who entered the trial properly accounted for at its conclusion?	Yes	Yes	Yes	Yes	Yes	Yes
4. Were patients, health workers and study personnel 'blind' to treatment?	No	No	Yes	No	No	No
5. Were the groups similar at the start of the trial	Yes	Can't tell	Yes	Yes	Yes	Yes
6. Aside from the experimental intervention, were the groups treated equally?	Yes	Yes	Yes	Yes	Yes	Yes
8. How precise was the estimate of the treatment effect?	Very confidence intervals mentioned 6-month 95% CI: 0.01, 0.02; 12-month 95% CI: 0.01, 0.01).	Not CI mentioned	No CI mentioned	Very CI mentioned	No CI mentioned	Very CI mentioned

9. Can the results be applied to the local population, or in your context?	Yes	Yes	Yes	Yes	No	Yes
10. Were all clinically important outcomes considered?	Yes	Yes	Yes	Yes	Yes	Yes
11. Are the benefits worth the harms and costs?	Yes	Yes	Yes	No	Yes	Yes
